# Nematic Liquid Crystal on a Two Dimensional Hexagonal Lattice and its Application

**DOI:** 10.1038/srep13331

**Published:** 2015-08-20

**Authors:** Muhammad Arslan Shehzad, Dung Hoang Tien, M Waqas Iqbal, Jonghwa Eom, J. H. Park, Chanyong Hwang, Yongho Seo

**Affiliations:** 1 Faculty of Nanotechnology & Advanced Materials, HMC, and GRI, Sejong University, Seoul 143-747, South Korea; 2 Department of Physics and Graphene Research Institute, Sejong University, Seoul, Korea; 3 Department of Materials Science and Engineering, Chungnam National University, Daejeon 305-764, South Korea; 4 Center for Nanometrology, Korea Research Institute of Standards and Science, Yusong PO Box 102, Daejeon 305-600, Korea

## Abstract

We have studied the alignment of liquid crystal adsorbed onto graphene and hexagonal boron nitride by using a polarized optical microscope. From the experimental data, it was found that there were 6 different alignment orientations of the liquid crystal molecules on a single crystal substrate. This result has never been reported and is quite different from other previous results. As the hexagonal lattice has a threefold rotational symmetry, three different alignment orientations were expected, but our result seems counter-intuitive. We explain this result considering the bending of the tail of the liquid crystal molecules. Using this anchoring effect with six accurate discrete angles, a novel non-volatile display can be developed with micron-scale pixel size, due to the molecular level accuracy of the alignment.

Liquid crystal (LC) has a unique feature of its natural appearance of a long-range orientational arrangement. LC can be used in numerous applications because of its birefringent behavior. LC has ability to change the orientation of molecules in response to very weak physical or chemical interactions, such as an electric field, surface modifications and pressure gradients^1^. LC can be aligned using polymers^2^, crystalline surfaces^3^, and patterned surfaces^4^. It is known that the orientational arrangement of LC is dependent on the van der Waals interaction with substrates. Recent findings of 2D materials as graphene and boron nitride provoked the interest of researchers due to their potential applications. Conventional chemical vapor deposition (CVD) technique is usually employed to synthesize graphene^5^ and boron nitride^6^ films. Boundaries between the grains, defects and grain size of these materials have a great influence on the electronic and mechanical properties^7^. In order to observe these defects and grain orientation, researchers have used such different equipment as transmission electron microscopy^8^, atomic force microscopy (AFM)^9^, and scanning tunneling microscopy^10^. These techniques usually need thorough sample preparation and dedicated control on equipment.

Recently, Kim *et al.*^11^ developed an interesting technique to visualize domain and grain boundaries of graphene using birefringence of liquid crystal. They reported that liquid crystal molecules were found to be aligned along crystalline orientations of the graphene as a consequence of strong epitaxial interactions between liquid crystal molecules and graphene^11^. More recently, Son *et al.*^12^ discussed the phase transition using the same technique to determine the defects in CVD grown graphene during the transfer process. Although their experimental findings have great influence on determining the grain orientation and defects without complex processes, neither investigated detailed preferential interaction between LC and graphene.

As it is already known, nematic LC on graphene or highly ordered pyrolytic graphite (HOPG) has specific preferential alignments. The crystal structure of graphene has a hexagonal lattice, and there are three arm-chair edge directions with a 120° interval. Thus, liquid crystal molecules on a single crystalline graphene tend to align along one of the three orientations^13^, and the LC molecules with the same orientation form a domain. At the domain boundaries, abrupt directional change occurs. Also, LC director orientation exhibits a specific relation with the graphene edges^14^. In the case of armchair edges, the LC director exhibits the same orientation or is rotated with a deviation of ±60° from the edge. Zigzag edges are oriented at a ±30° deviation from the arm-chair edges. The anchoring force that aligns the LCs into a planar configuration increases with increasing numbers of graphene layers^15^.

In this work, we employed Kim’s method^11^ to visualize the domain orientation and boundaries on different materials, i.e., HOPG and hexagonal boron nitride (hBN), and studied the preferential alignment directions of liquid crystal on them. Interestingly, it was observed that liquid crystals align in six preferential orientations on a single crystal, instead of three. We explain this unexpected result considering the geometry of the LC molecule, and discuss its potential application for a novel liquid crystal device (LCD).

## Results and Discussion

In order to determine the molecular interaction between LC and the surface, polarization effect of the LC was observed in graphite and hBN. HOPG was exfoliated once with Scotch tape and was transferred to a glass substrate. To determine the roughness of the exfoliated HOPG, AFM was employed and the surface was found to be smooth (Refer to supplementary Fig. S4(d)). Raman spectroscopy was also adopted, which clearly revealed the presence of a graphite layer on the glass substrate (Refer to supplementary Fig. S4(c)).

**Figure 1:**
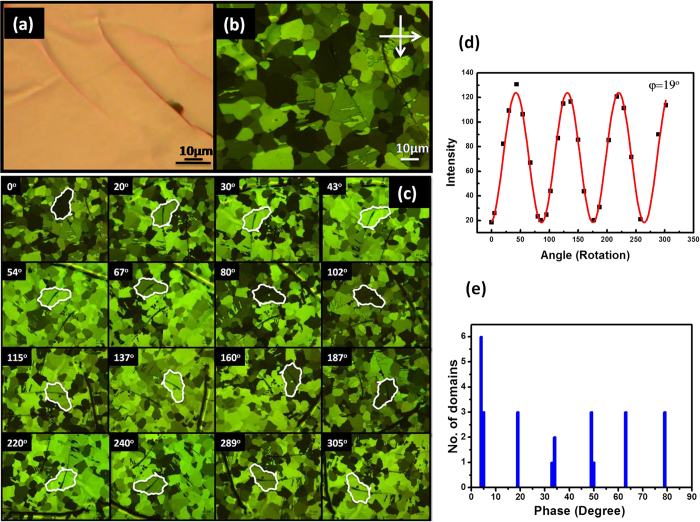
Liquid crystal alignment on HOPG surface. HOPG was exfoliated and transferred to a glass substrate. Liquid crystal was then spin-coated on the same sample. (**a**) Optical microscope image of HOPG without polarizer (**b**) POM with cross-polarizer image shows LC domains with clear boundaries. (**c**) The sample was rotated in clockwise direction. The domain marked was dark at 0^o^, which became bright at 30^o^. (**d**) Intensity versus rotation angle was plotted and fitted into a sinusoidal function. (**e**) The phases for more domains were measure, and the number of domains with the same phase was plotted.

Nematic liquid crystal (4-pentyl-4-cyano-biphenyl, 5CB) with a clearing point of 33 ^°^C was used to visualize the domains of transferred films. LC was spin-coated on HOPG. The sample was then heated above the clearing point to the isotropic phase and then slowly cooled down to the nematic phase. Exfoliated graphite was marked with mechanical scratches and observed with an optical microscope (Fig. 1(a)). A polarized optical microscope (POM) with perpendicular alignment between polarizer and analyzer was used to study the interaction between graphene and liquid crystal. The POM image showed different alignments of LC on the single crystal graphite surface (Fig. 1(b)). The LC domains having the same contrast were observed, and domain boundaries were clear under POM. It was known that the LC domain has strong correlation with grain of CVD grown graphene. Grains of CVD grown graphene on copper foil were also observed using the same liquid crystals (Supplementary Fig. S3). As LC domains have different polarization angle, they have different brightness. The sample was rotated in the clockwise direction, and the POM images were taken as shown in Fig. 1(c). Consecutive 25 images at different angles were taken to complete the full rotation. The brightness change as a function of the angle was observed for more than 20 domains. A representative domain was marked in Fig. 1(d), where one can see that the brightness was oscillated as a sinusoidal function of the rotation angle. The brightness of each grain was calculated using MATLAB (See Methods). The calculated intensity for the domain was plotted with rotation angle, as shown in Fig. 1(e), and it was observed that four cycles were repeated within the whole rotation. Fitting was done using the following equation in order to get the phase or orientation angle of liquid crystal:


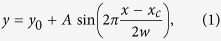


where 2*w* is period (90^o^), *A* and *y*_*0*_ are fitting parameters and, *x*_*c*_ is the phase. A histogram in Fig. 1(e) shows the calculated phases for 25 grains, where the x-axis represents the different phase angles and the y-axis represents the number of domains corresponding to the phases. Interestingly, it was observed that six phase angles were preferred with 15^o^ ± 1^o^ spacing. In order to confirm the reliability of hypothesized result, the experiment was repeated and the same result was obtained. Comprehensive raw data of the process are shown in supplementary Fig. S2. It was claimed that there were six discrete angles at which the LC molecules tend to anchor graphite surface. Note that two LCs (5CB) purchased from different companies showed the same results (Supplementary Fig. S1).

**Figure 2:**
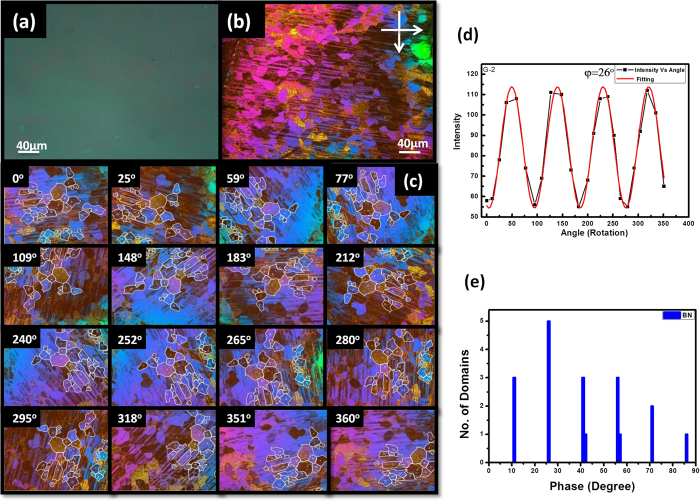
Liquid crystal alignment on hexagonal boron nitride surface. CVD grown hBN was transferred to silicon substrate. (**a**) Optical image of transferred hBN. Liquid crystal was then spin coated on the hBN. (**b**) POM image with cross-polarizer of hBN coated with liquid crystal. (**c**) Sample was rotated in clockwise direction, where different domains were marked on the images. (**d**) Intensity versus rotation-angle was plotted and fitted to extract the phases. (**e**) Histogram of orientation angle versus number of grains shows six discrete angles preferred.

The same experiment was performed on a different substrate. Highly oriented hBN was synthesized on a polished copper foil using the conventional CVD technique (See Methods). The copper foil was electro-polished and heated up to 900 ^°^C, and borazine and hydrogen gases were used to synthesize hBN. (Supplementary Fig. S5). Grown hBN was then transferred to a silicon substrate using the standard wet transfer method. An optical image confirmed that BN was completely transferred to the silicon substrate (Fig. 2(a)). The Raman spectrum of the hBN was measured, which showed a broad peak at 1369 cm^−1^ (Supplementary Fig. S6(c)), confirming few-layer hBN was grown with high crystallinity. The surface roughness of transferred hBN was estimated using AFM, and the surface was found to be smooth with no remarkable features (Supplementary Fig. S6(d)). Liquid crystal was then spin-coated on the hBN, and POM with cross-polarizer was used to produce the image as shown in Fig. 2(b). This was the first observation of the LC alignment along the hBN crystallographic angle. This image shows continuously varying colors differently from the HOPG result, which is attributed to the non-uniformity of the thickness of LC. Due to the interference between the beams reflected from the top surfaces of LC and hBN, continuously changing colors appeared depending on the thickness. The sample was rotated in the clockwise direction, and different domains were marked as shown in Fig. 2(c). Intensity versus rotation angle was plotted and fitted to Eq.(1) in order to get the preferred orientation angle, as shown in Fig. 2(d). The number of domains having the same phases is shown in a histogram (Fig. 2(e)). It shows six discrete angles with 15^o^ spacing, which were preferred for LC to anchor the hBN surface, as was the case with graphite. This also confirms that the behavior of six preferential orientations is not only for graphite but it occurs to other hexagonal lattices. It was established that this type of interaction is purely molecular instead of the surface morphology^11^. To confirm the molecular interaction between hBN and LC, an a small flake of hBN with a smooth surface was exfoliated and mounted on a silicon substrate and coated with LC. The POM image confirmed the presence of different domains with the molecular alignment of liquid crystals on hBN. The images of the rotated sample at different angles showed the same behavior, which confirms the LC-hBN alignment was mainly based on the molecular interaction, instead of the surface morphology like ripples or a grain boundary. (Supplementary, Fig. S7).

**Figure 3:**
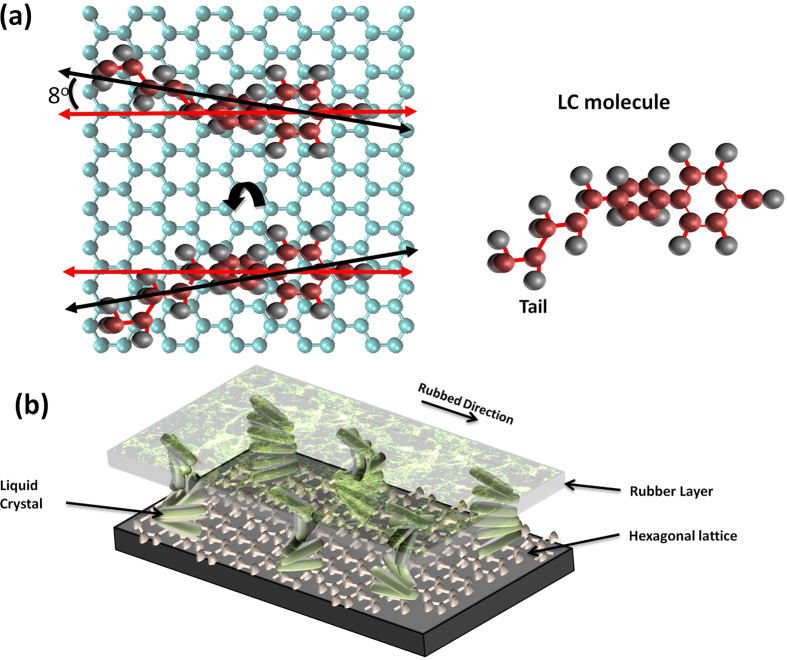
Schematics of liquid crystal alignment on hexagonal lattice. (**a**) Schematics of a LC molecule adhered on hexagonal lattice. The tail of the 5CB LC molecule is slightly titled (~8^o^) from the axis of the body composed of two benzene rings as shown in black line. As the molecule can be flipped 180^o^ with the same probability, two different polarization directions are induced with ~15^o^ deviation. As three armchair edge directions have two flipping degeneracy, 6-fold symmetry was found. (**b**) Schematics of LC cell with unidirectional rubbed layer. Direction of alignment of LC molecules on the hexagonal lattice (bottom layer) is selected among six preferred orientations. The unidirectional rubbed alignment layer is placed at the top, so that LC molecules will be twisted depending on the angle differences, and the twisted LC layer reflects different color depending on the twist rate.

Our result of the six aligning directions, instead of three, is different from recently published results^13^. One may consider the 3 zig-zagged edge directions additional to 3 arm-chair directions, but that would be double counting because they have 30^o^ difference. In order to understand the nature of six aligning directions, a schematic drawing with a possible configuration is shown in Fig. 3(a). A benzene ring in the LC molecule (5CB) adsorbs on the substrate being coherently aligned^11^. Previously, it was suggested that van der Waals forces between alkyl groups plays a role in the alignment of LC molecules on the hexagonal substrate^11^. There are two different anchoring preferences, as the LC molecule can be flipped 180^o^. The tail of the molecule is slightly titled (~8^o^) from the hexagonal crystalline axis as shown in the black line in Fig. 3(a)^16^. When the molecule is flipped upside down, the tilt angle will be in the opposite direction where the difference between orientations would be about 15^o^. As all three different aligning orientations (arm-chair edge direction) have two degeneracy, there must be six directions. It was confirmed that the six peaks with equal spacing was not found when a different type of LC was used. (See Figure S10)

**Figure 4:**
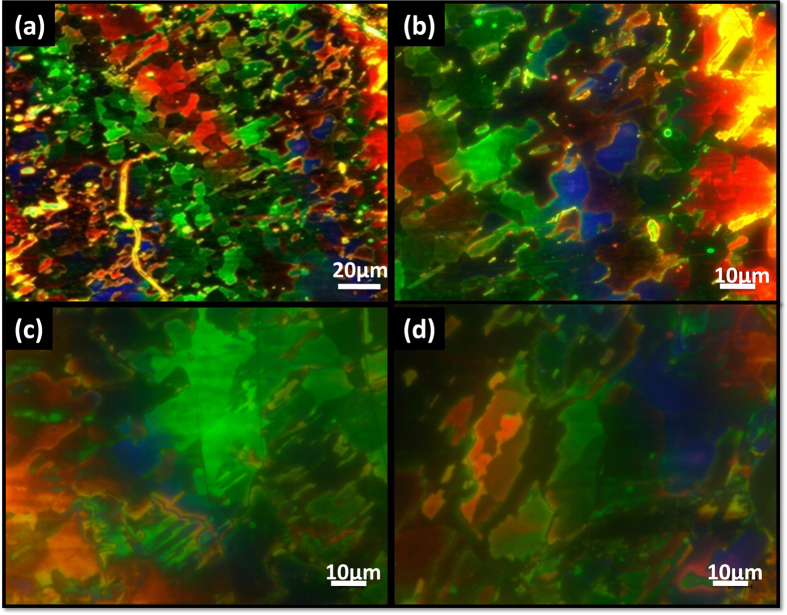
Optical microscopic images of the LC device between HOPG and rubbed layer. (**a**) Low magnification images confirm the presence of more than three colors including red, green and blue. (**b–d**) High magnification images of different positions of the same sample show many LC domains having different colors within a single-crystal substrate.

The LC device was fabricated as shown in Fig. 3(b). HOPG on glass was spin coated with liquid crystal and a thin glass with unidirectional rubbed polyimide layer was placed on LC so that molecules will aligned unidirectional at the LC/polyimide interface. Alignment of the LC molecules in bottom side was dependant on the crystalline axis and LC preferential directions. The LC thickness was kept ~5 μm using spacers. Expected configurations of the LC directors are shown in Fig. 3(b) where the LC directors are twisted and a photonic band gap^17^ is induced. Optical images of HOPG/LC domains on the device are showed in Fig. 4 with low and high magnifications. The domains have different colors, which originated from different photonic band gaps corresponding to twisting angles due to the Bragg condition^18^, as well as a thickness gradient of LC. High magnification images (Fig. 4(b–d)) of different positions show that each domain with a discrete boundary has different color, confirming that the six preferential directions of LC exist in this device.

**Figure 5:**
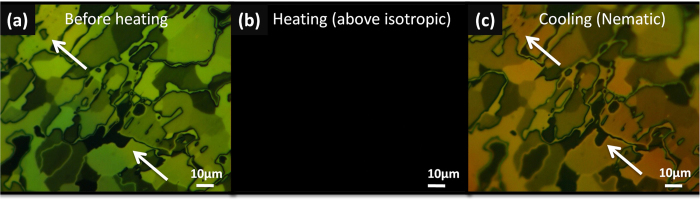
Thermal stability of liquid crystal on HOPG. (**a**) POM image was obtained after LC was deposited on HOPG. (**b**) Sample was heated above the clearing transition temperature (33 ^°^C), and POM image became dark, confirming LC was in isotropic phase. (**c**) After it was cooled again, most LC molecules were aligned with the original shapes of domains, but interestingly some of domains was created or annihilated, as pointed by arrows.

The thermal stability of LC between graphite and rubbed layer was studied. A sandwich structure cell was fabricated with a 5 μm gap and was filled with LC by capillary action. When sample was heated (60 ^°^C) above the isotropic transition temperature, the POM image revealed dark color due to the random orientation of liquid crystal (Fig. 5(b)). Then, it was cooled down to the nematic phase again. Different background colors between the images before (Fig. 5(a)) and after (Fig. 5(c)) heating is due to the slight change of the thickness of LC. It was observed that most LC domains were recovered, confirming the anchoring orientation of LCs on graphite surface was not reoriented during the thermal treatment. However, the arrows in the Fig. 5 indicate an exception where the domain boundary was moved, and domains disappeared and were generated during the thermal cycle. This indicates that the aligning direction of the LC molecules was not strictly determined by the morphological structure of the substrate, but the LC molecules have freedom to change their anchoring orientation to a certain degree.

## Conclusion

In this investigation, we analyzed the alignment of liquid crystal on different hexagonal lattice surfaces. We have found six preferential directions for the spontaneous alignment of liquid crystals on graphene, graphite and hBN. Judging from the clear discrete angles, the alignment is on molecular scale, that is, the benzene ring of liquid crystal is coherently aligned on the hexagonal lattice. This study suggests a principle to develop a novel non-volatile display having micron-size pixels. Realization of this hexa-stable display device could have crucial advantages compared with the conventional LC display (LCD): (1) non-volatility, (2) no need to have backlight unit, (3) high definition pixels. Especially, ultra high resolution pixels can be realized, as the alignment of LC director works by molecular interaction.

## Methods

### Synthesis of Boron Nitride and HOPG

The hBN film was grown on Cu foil (Alfa Aesar, 99.8% pure, 25 μm thick) using thermal CVD. The mechanically polished and electro-polished Cu foil was annealed at 990 ^°^C for 30 min with H_2_ gas at a flow rate of 5 sccm. Ammonia borane (Sigma-Aldrich, 97% pure) was used as a precursor, which was thermally decomposed into hydrogen, aminoborane, and borazine at a temperature range from 80 to 120 ^°^C. After annealing, hBN was synthesized with borazine gas and hydrogen at 997 ^°^C for 30 min. The furnace was cooled from 997 to 500 ^°^C at a rate of ~35 ^°^C/min. The flow rate was kept quite slow in order to get a highly oriented BN surface. Films were transferred to the substrates i.e., silicon or glass using the standard wet transfer method using poly(methyl methacrylate) (PMMA). Commercially available HOPG was exfoliated once with Scotch tape and transferred to the substrates.

### LC Alignment

Commercially available nematic liquid crystals (5CB, Sigma Aldrich and Qingdao Intermodal Trading Co. Ltd.) were used. 0.5 μl liquid crystal was spin-coated (3000 rpm) for 60 s on CVD grown hBN and exfoliated HOPG films to get ~1 μm thick layer. Liquid crystal was aligned spontaneously on the surfaces and domain orientation, and boundaries were observed under an optical microscope with a cross polarizer. Heating was done in order to observe the stability of interaction throughout the phase transitions. After samples were observed at room temperature, the temperature was raised to 60^o^ above the clearing point for 15 minutes, followed by cooling in ambient air. For electro-optic experiments, polyvinyl alcohol (PVA) was spin-coated on glass slide at 3000 rpm. The film was rubbed in one direction and was placed over the LC coated sample. The cell gap (~5 μm) was controlled using thin tape and micro beads between two sides.

### Characterization

The LC alignment with domains was observed using POM (Olympus, BX-51). The polarizer and analyzer were oriented perpendicular to each other. Raman spectroscopy with 514 nm laser (Renishaw micro-Raman System) was used and power was kept below 1.0 mW to avoid laser-induced heating. Topography was measured in tapping mode using a commercial AFM (NanoFocus, n-Tracer).

### Analysis

**Figure 6:**
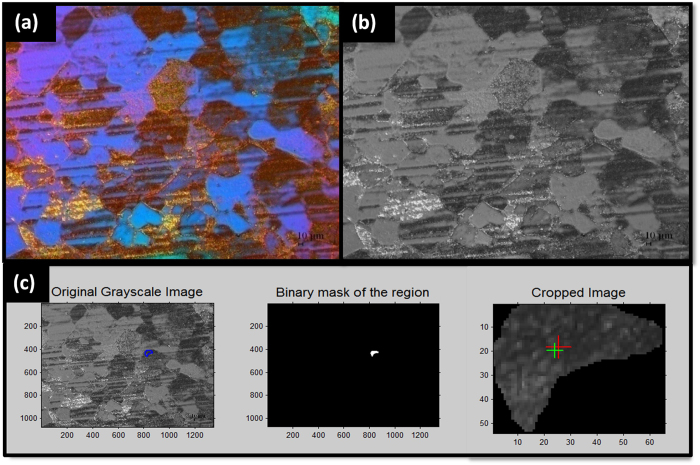
Calculation of the mean intensity values for colorful domains. (**a**) POM original image measured from a CMOS camera was color-coded. (**b**) The color image was converted into grayscale. (**c**) From a freehand drawing of a selected domain, a binary mask was generated. The image was cropped using the binary mask, and the mean intensity of pixels of the area was calculated.

Original POM image obtained from a CMOS camera in Fig. 6(a) was color-coded. In order to extract the intensity of colors, the RGB color image was converted into gray-scale image, as shown in Fig. 6(b). Particular domain area was selected using freehand drawing and coordinates of the boundary was recorded in a binary mask (Fig. 6(c)). The image was cropped using the mask, and the mean intensity of cropped pixels was calculated. MATLAB software was used for this data processing.

## Additional Information

**How to cite this article**: Arslan Shehzad, M. *et al.* Nematic Liquid Crystal on a Two Dimensional Hexagonal Lattice and its Application. *Sci. Rep.* **5**, 13331; doi: 10.1038/srep13331 (2015).

## Supplementary Material

Supplementary Information
