# Supplementary material for: Nematic Liquid Crystal on a Two Dimensional Hexagonal Lattice and its Application
Source: Sci Rep. 2015 Aug 20;5:13331. doi: 10.1038/srep13331 (PMC4542626; doi:10.1038/srep13331)
Supplement: Supplementary Information [file srep13331-s1.pdf]

Nematic Liquid Crystal on a Two Dimensional Hexagonal Lattice and its Application

Muhammad Arslan Shehzad[1], Dung Hoang Tien[1], M Waqas Iqbal[2], Jonghwa Eom[2],

J. H. Park[3], Chanyong Hwang[4], Yongho Seo[1,*]

[1]Faculty of Nanotechnology & Advanced Materials, HMC, and GRI, Sejong University, Seoul 143-747, South Korea
[2]Department of Physics and Graphene Research Institute, Sejong University, Seoul, Korea
[3]Department of Materials Science and Engineering, Chungnam National University
[4]Center for Nanometrology, Korea Research Institute of Standards and Science, Daejeon, Korea

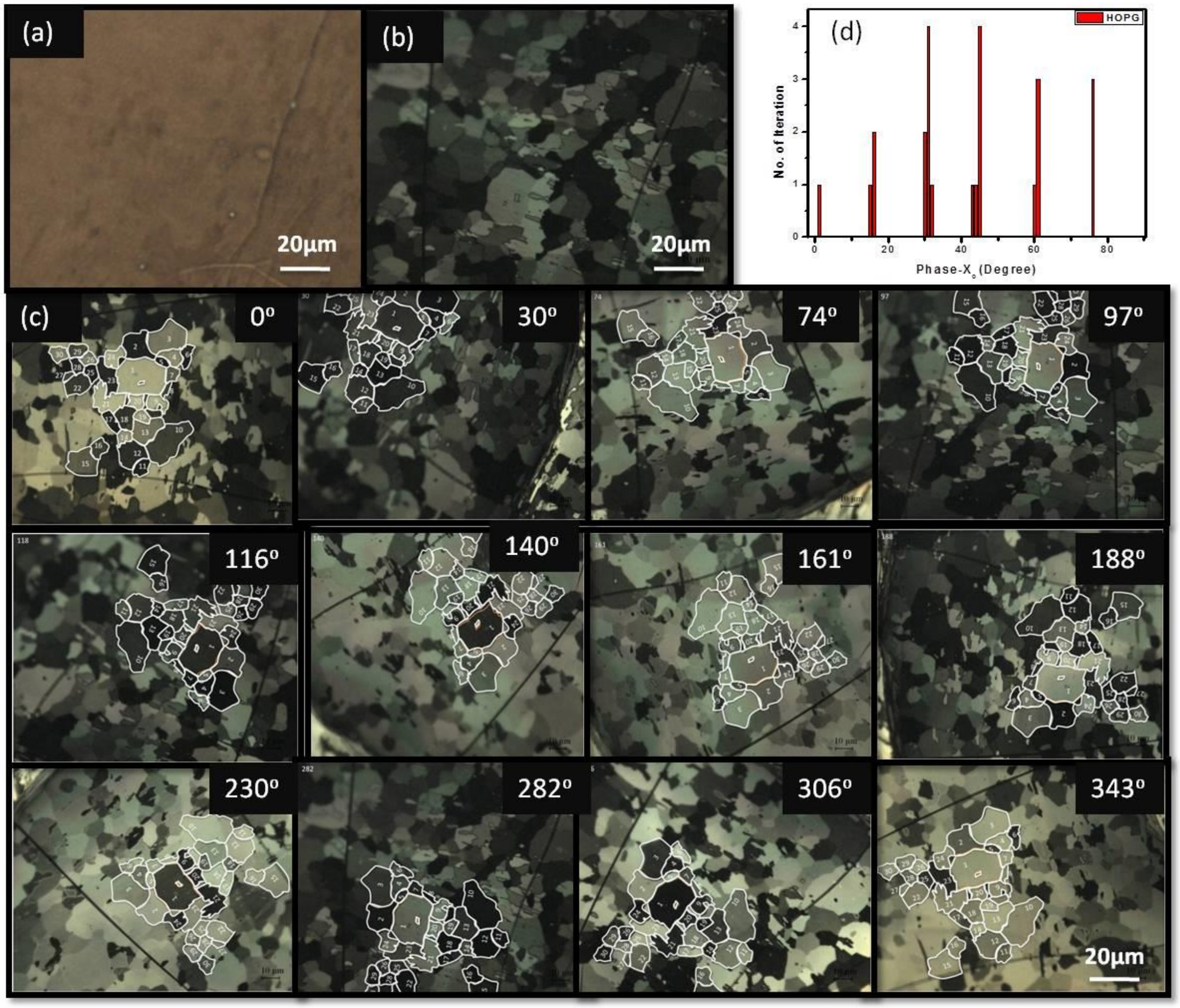

Figure S1: 4-pentyl-4-cyano-biphenyl (5CB) from different company was used in order to confirm the alignment. (a) and (b) show optical images with and without LC (Qingdao Intermodal Trading Co. Ltd.). (c) Different domains were marked and counter clockwise rotation was done and intensity of each domain was estimated, and phase was calculated. (d) Phase vs. intensity plot confirmed six discrete orientations of 5CB on HOPG.

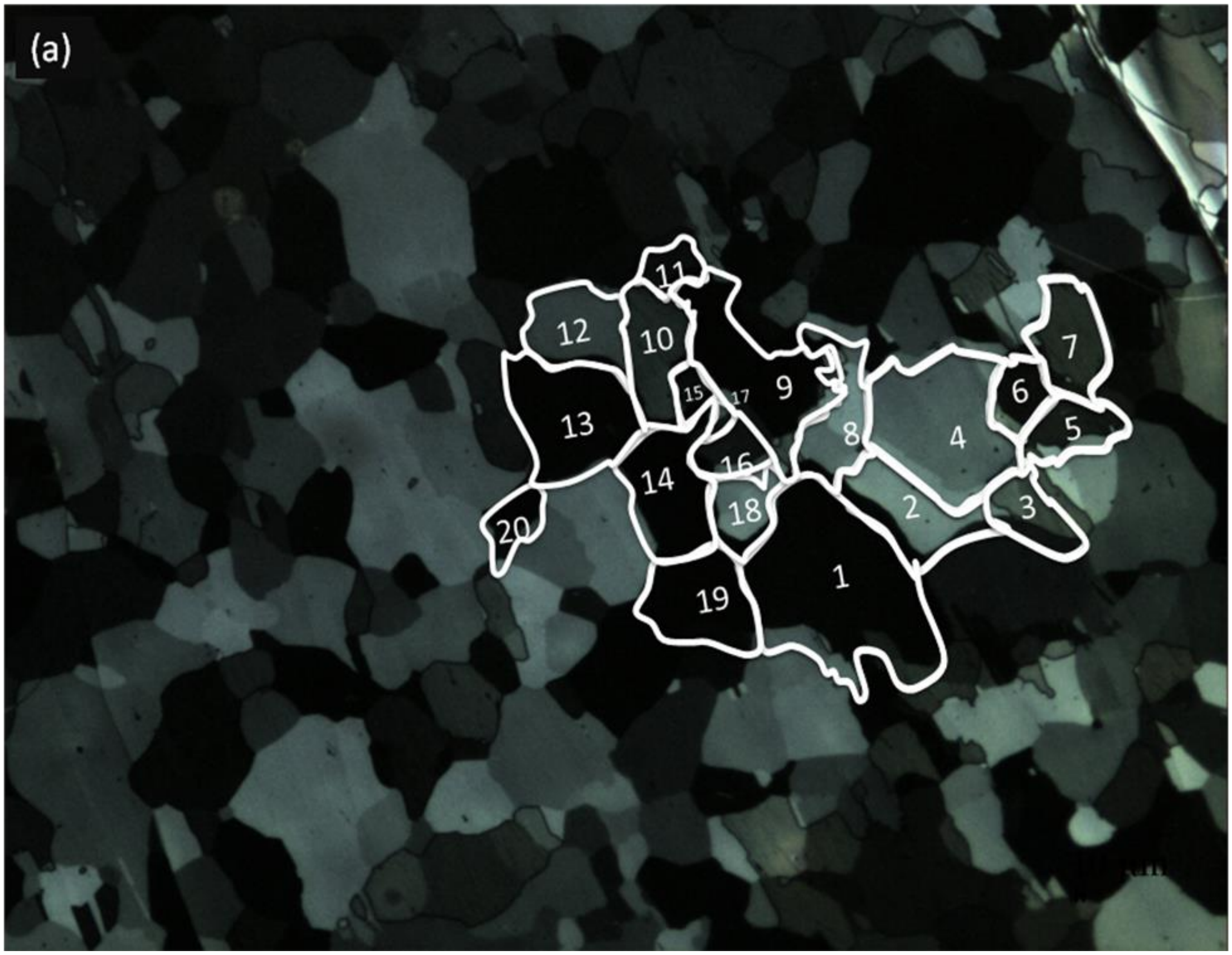
(a)
11
12
10
7
15
17
9
6
13
8
4
5
16
14
2
3
18
20
1
19

(b)

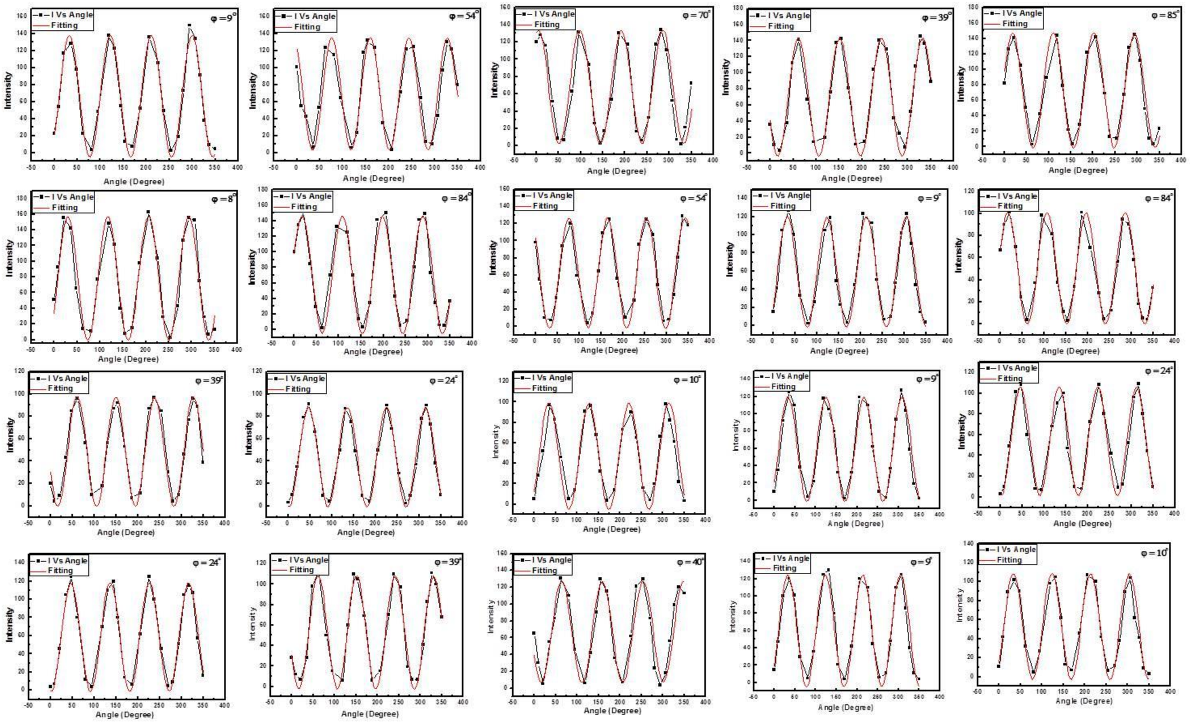

(c)

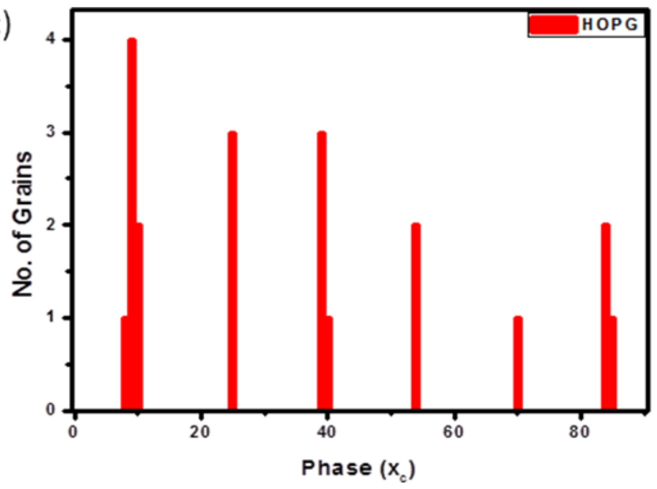

Figure S2: Intensity vs. rotation angle. (a) Different domains in POM image was marked with independent numbers. (b) 20 domains were selected and intensity was plotted against rotational angle. Fitting was done in order to get phase of each domain. It was observed that the domain No: 1,6,9,13,14,19 and 20 have darkest parts with phase of ~9°, and similarly 2nd and 8th were brightest with phase of 54° (c) Histogram shows the number of domains having the same phase.

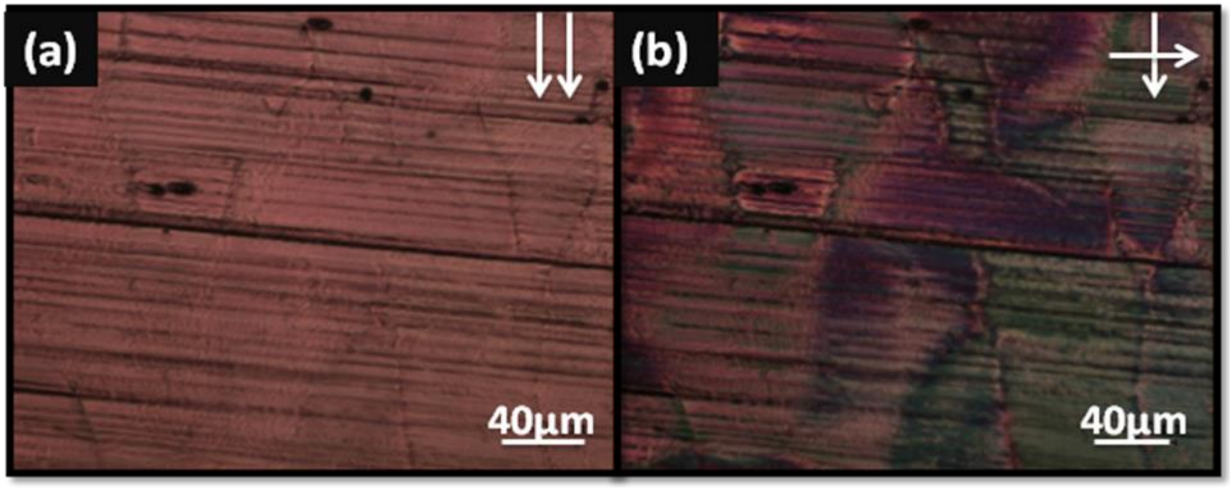

Figure S3: Direct observation of graphene domains on copper. Polarized optical microscope images of graphene grown on copper with (a) parallel and (b) cross polarizer/analyzer clearly show different color and contrast.

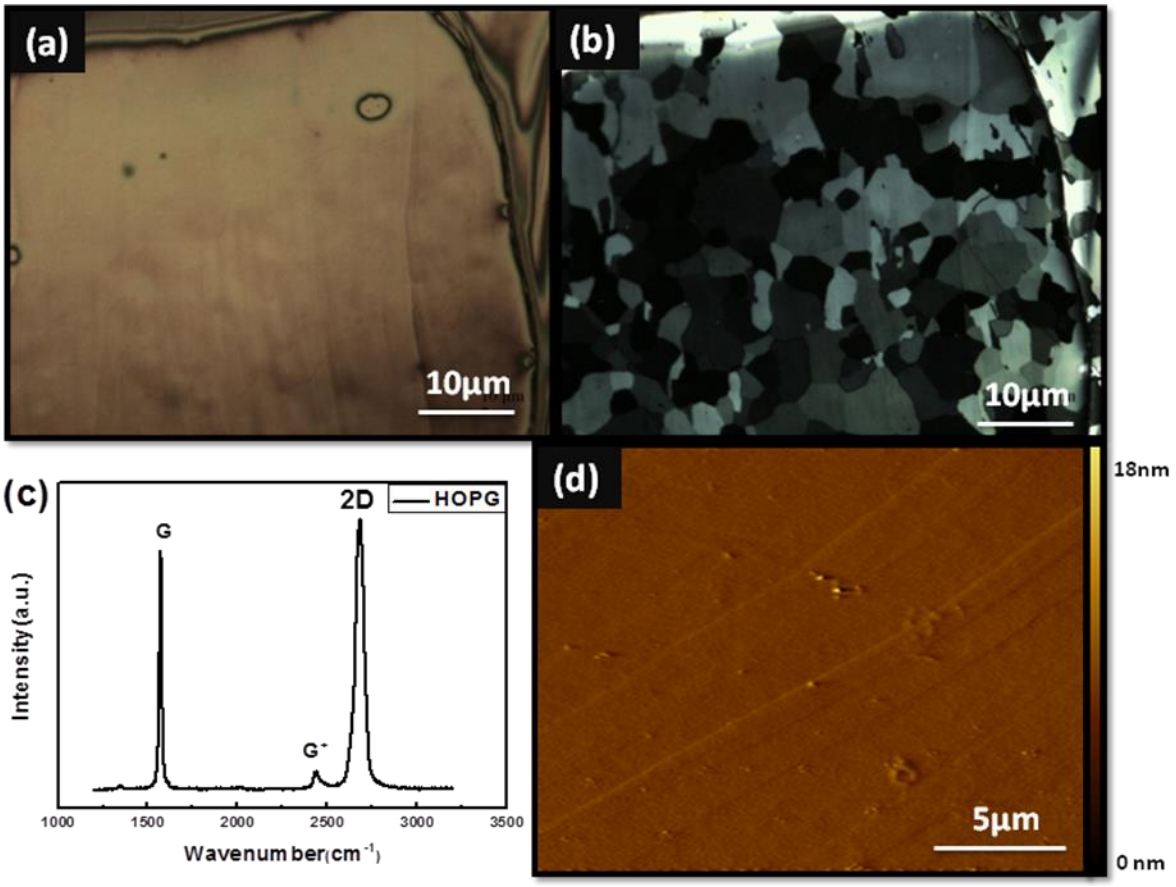

Figure S4: Exfoliated HOPG was observed with POM (a) with and (b) without liquid crystal. (c) Raman spectrum of HOPG with 514 nm laser with very low D peak around 1350 cm^{-1} confirms the surface of highly crystalline defect-free graphite. (d) 20×20 μm^2 AFM scan of HOPG in contact mode, in ambient air was done in order to inspect the surface topography of exfoliated sample. It was revealed that transferred HOPG was almost defect-free with smooth surface.

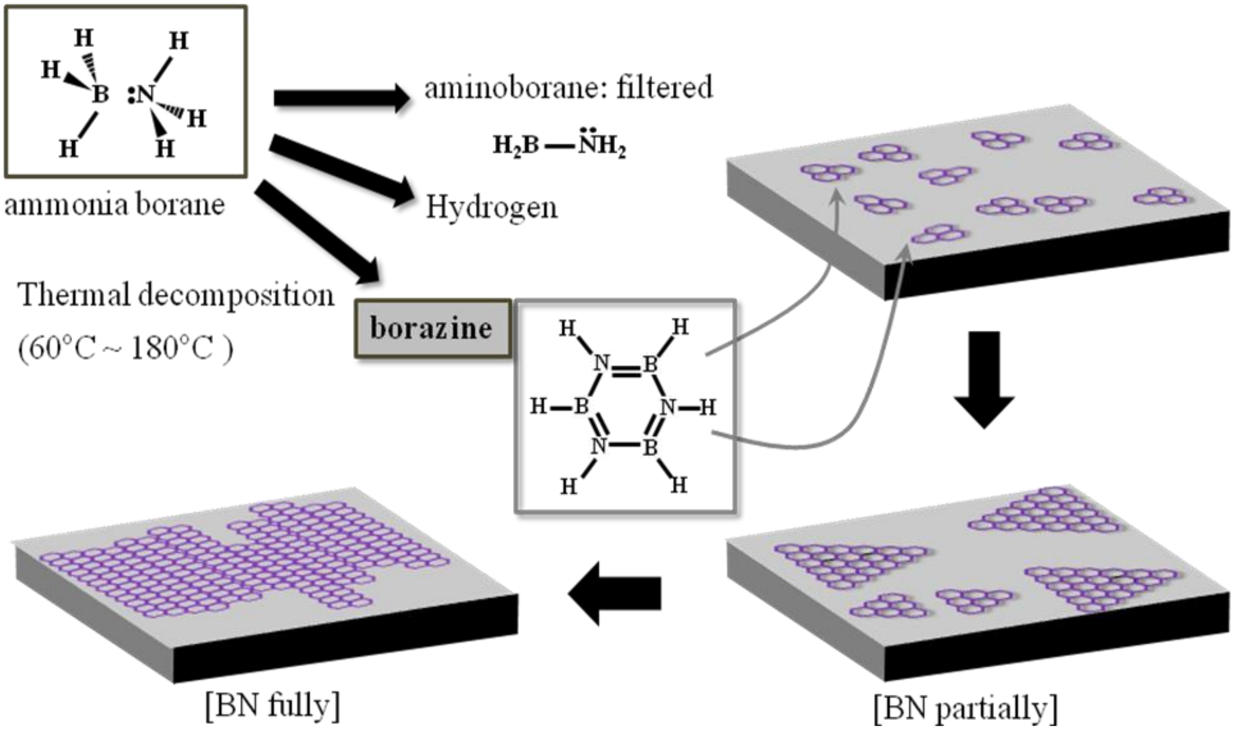

Figure S5: Schematics of CVD-Boron Nitride on electro-polished Copper. Cu foil was annealed at 990 °slow rate. Ammonia borane was thermally decomposed into hydrogen, aminoborane, and borazine at a temperature range from 80 to 120 ℃. After cleaning, h-BN was grown with borazine gas and hydrogen at 997 ℃ for 30 min.

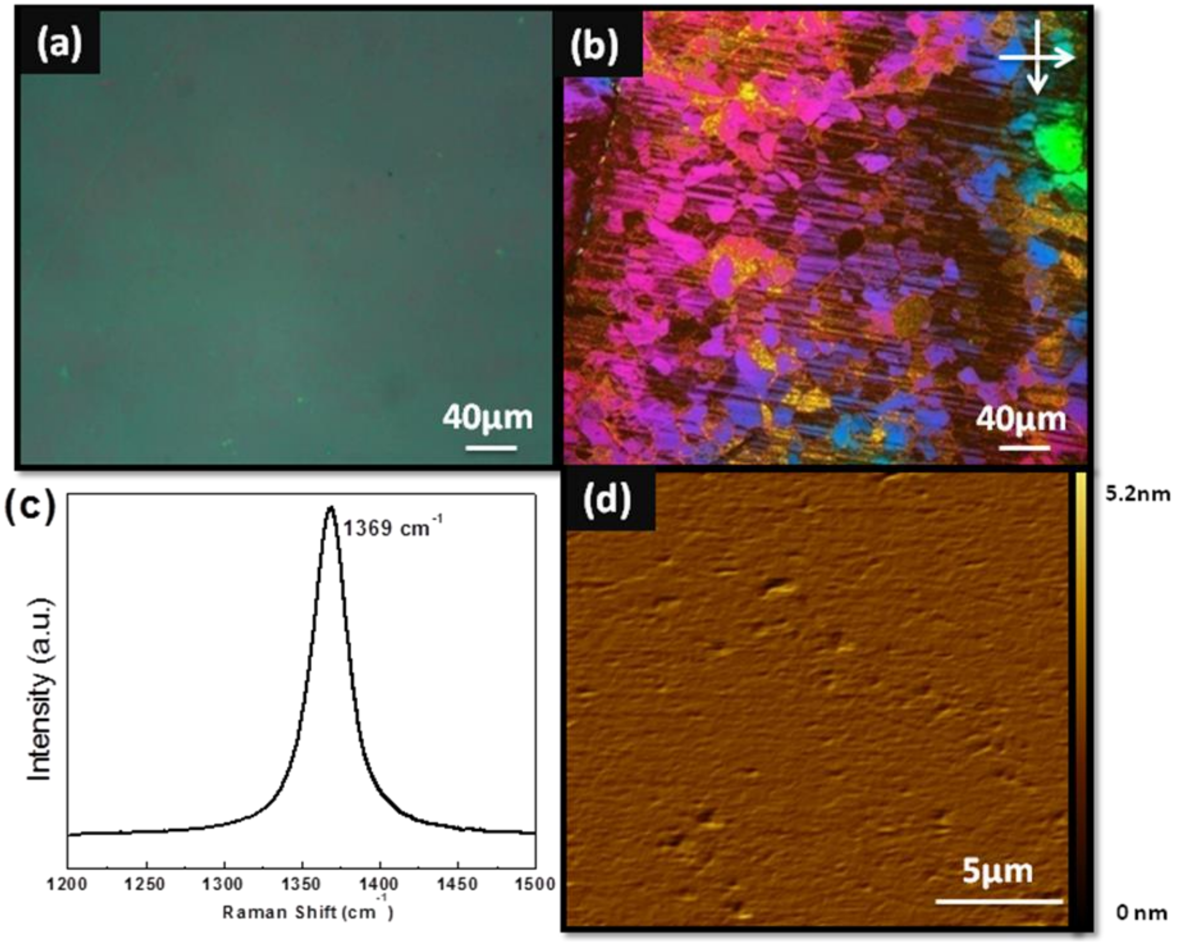

Figure S6: (a) & (b) Optical image of transferred CVD-BN film on silicon substrate. (b) shows POM image of transferred BN with liquid crystal. Clearly we can observe domain boundaries and different colors which attributes to different orientational alignment of LC molecules. (c) Raman spectra of CVD BN showed broad peak at 1369cm^{-1} , shift was observed from bulk crystal of BN which attributes the presence of few layers of CVD-BN. (d) 20×20μm AFM scan of BN in contact mode, in ambient reveal smooth topology of with quite few defects which may corresponds to polymer residue in the transfer process.

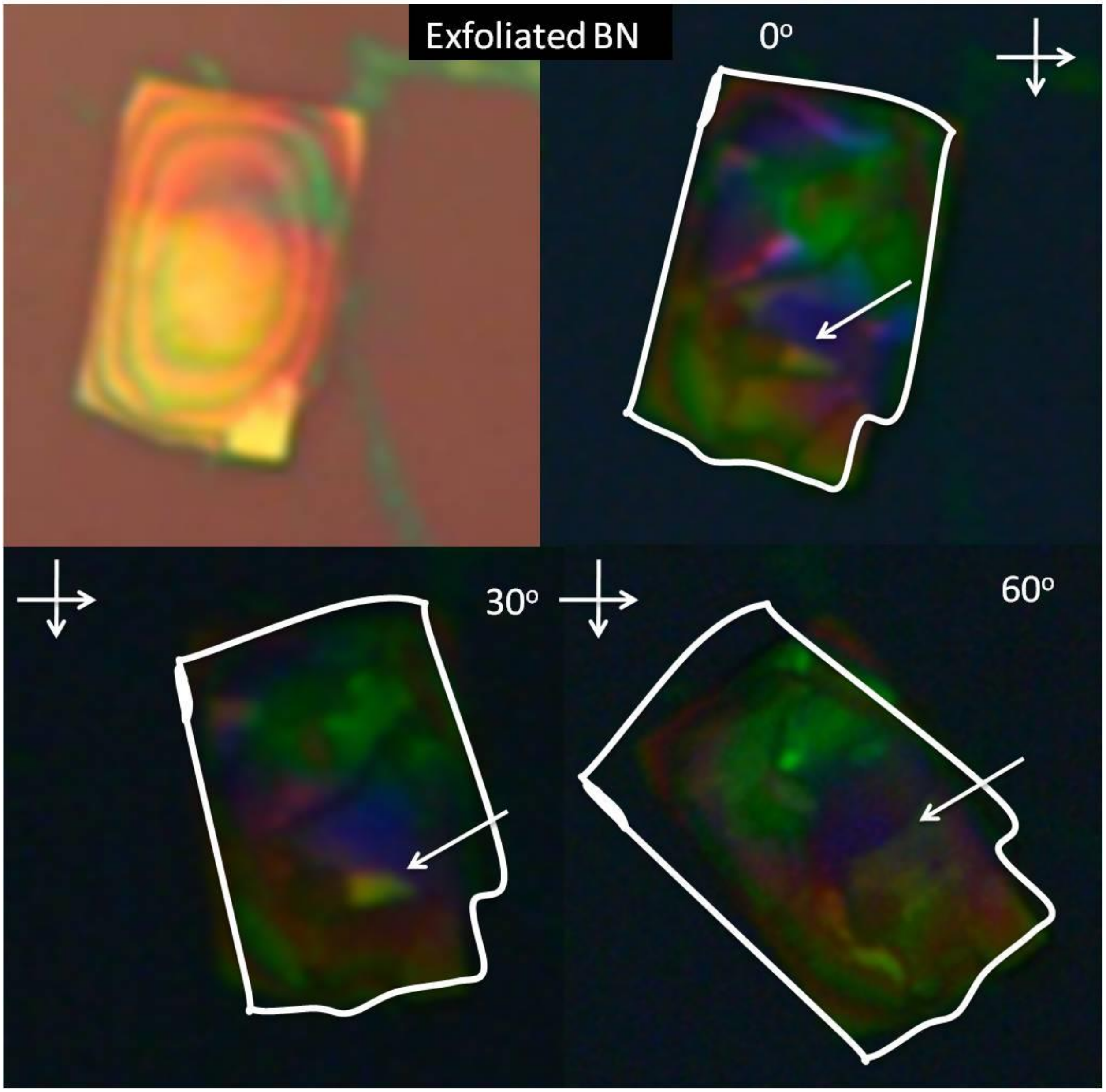

Figure S7: Molecular interaction between BN and liquid crystal, In order to confirm molecular interaction, an h-BN flake with smooth surface was exfoliated on silicon substrate and spin-coated with LC. (a) optical image of LC coated flake with no visible domain/boundaries (b) POM image further confirms the presence of different domains and molecular alignment of liquid crystals on BN. (c) & (d) Counter clockwise rotation confirms that some bright region was converted dark as molecular orientation become parallel to the polarizer or analyzer.

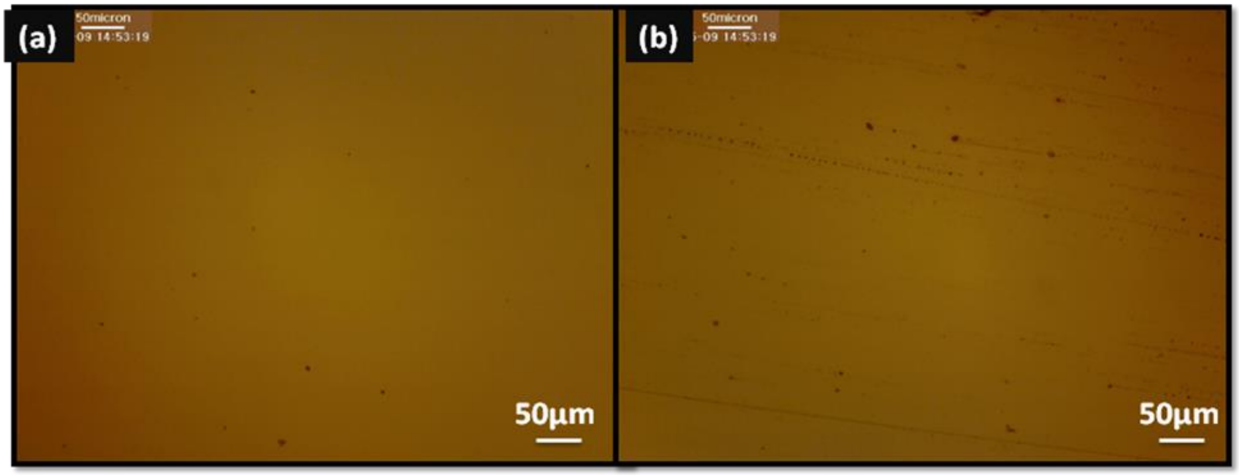

Figure S8: Rubbed alignment layer was used in order to observe the have clear birefringence. Polyvinyl alcohol (PVA) was spin coated on glass slide at 3000RPM in order to obtain ~2µm thick layer. (a) optical image of PVA film after spin coating (b) Film was unidirectional rubbed using smooth piece of cloth and was placed over the LC coated sample with spacers. Optical image clearly confirms the rubbing of thin film.

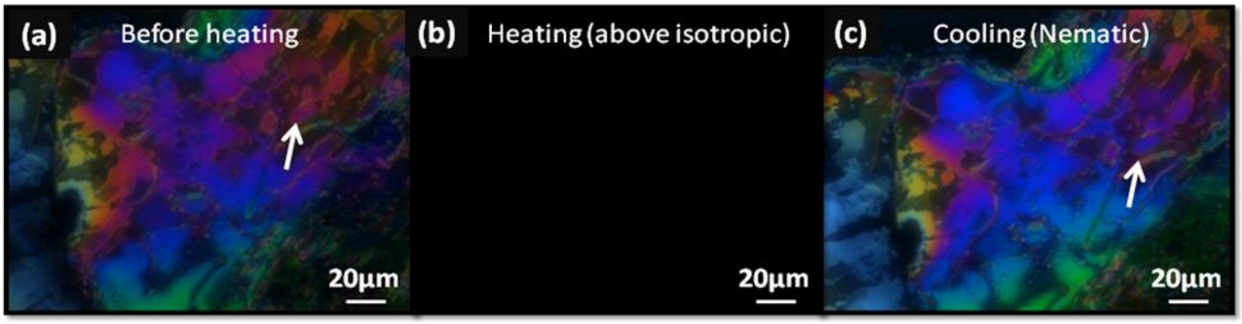

Figure S9: Thermal stability test, In order to confirm more than three symmetric orientations thermal stability test was done. After LC alignment, sample was heated above the isotropic transition temperature (60° C), POM image revealed dark color due to isotropic refractive index with random orientation of liquid crystal (b). Cooling was done in ambient in order to get nematic phase. It was observed that LC alignment on graphite surface was not completely recovered as some birefringent colors were different before (a) and after (c) thermal treatment. Arrow indicates red color of some domain was converted to blue after heat treatment. While neighboring domains with dark color remain consistent. This indicates that the aligned direction of LC molecules was not strictly determined by three phase symmetry as LC molecules can change their axis of rotation on external energy.

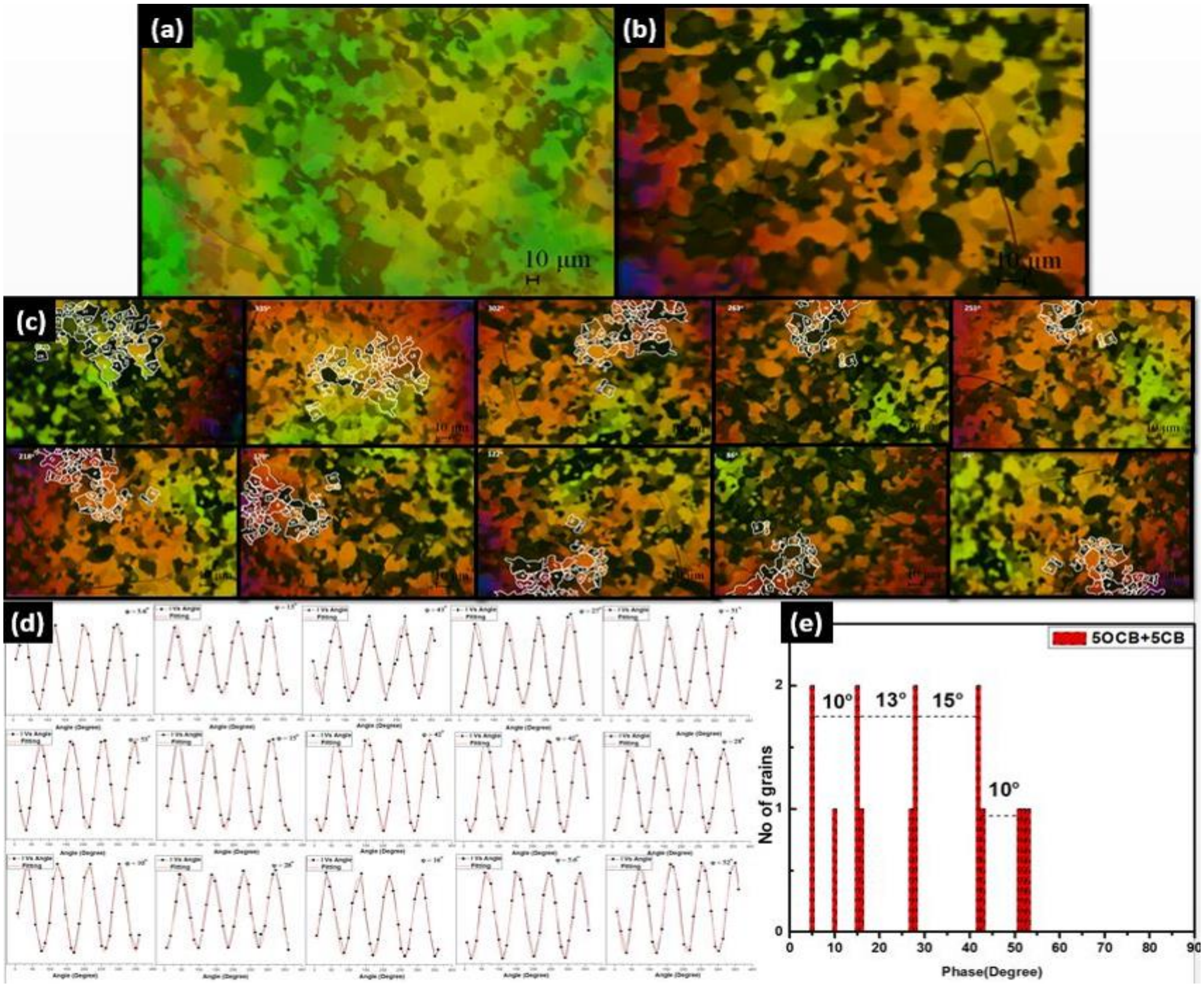

Figure S10: Liquid crystal (5CB+5OCB) alignment on highly ordered pyrolytic graphite (HOPG) surface. Low and high resolution POM images with cross polarizer further confirm the alignment with different domains and boundaries (a) & (b). (c) Analysis of different domains was done in reference to rotation and (d) Transmittance vs rotation-angle was plotted. (e) Phase vs number of grains show disorder in 15° intervals.

To confirm the universality of the six peak behavior, a different LC (5OCB, 4'-pentyloxy-4-biphenylcarbonitrile) having different molecular structure was tested for the same experiment. As nematic phase of 5OCB lies above the ambient so it was mixed with 5CB in order to use at room temperature. 5OCB+5CB (1:1) was spin coated on exfoliated HOPG sample, which was further heated to 80° C and subsequently cooled down to ambient in order to get nematic phase (a) POM image with cross polarizer further confirm the alignment with different domains and boundaries. (b) Shows high resolution POM with cross-polarizer image. (c) Different grains was marked and rotated in clockwise direction. Whole rotation-cycle was completed and

transmittance was estimated. (d) Transmittance vs rotation-angle was plotted, and fitting was done to more than fifteen grains in order to get the phase/orientation angle. (e) Phase vs number of grains shows hex-angle symmetry orientation was shifted from 15° of spacing to 13° and 10° which may be due to more tilt in the tail of liquid crystal. Although it is evident from the experimental data that bending of tail was shifted but further experimental work is required in order to demonstrate this effect.
